# Maelstrom Research guidelines for rigorous retrospective data harmonization

**DOI:** 10.1093/ije/dyw075

**Published:** 2016-06-05

**Authors:** Isabel Fortier, Parminder Raina, Edwin R Van den Heuvel, Lauren E Griffith, Camille Craig, Matilda Saliba, Dany Doiron, Ronald P Stolk, Bartha M Knoppers, Vincent Ferretti, Peter Granda, Paul Burton

**Affiliations:** 1 Research Institute of the McGill University Health Centre, Montreal, QC, Canada; 2 McMaster University, Department of Clinical Epidemiology and Biostatistics, Hamilton, ON, Canada; 3 Eindhoven University of Technology, Department of Mathematics and Computer Science, Eindhoven, The Netherlands; 4 University Medical Center Groningen, Department of Epidemiology, Groningen, Groningen, The Netherlands; 5 McGill University, Centre of Genomics and Policy, Montreal, Montrreal, QC, Canada; 6 Ontario Institute for Cancer Research, MaRS Centre, Toronto, ON, Canada; 7 University of Michigan, Inter-university Consortium for Political and Social Research (ICPSR), Ann Arbor, MI, USA; 8 University of Bristol, D2K Research Group, School of Social and Community Medicine, Bristol, UK

**Keywords:** Data harmonization, data integration, data processing, individual participant data, retrospective harmonization, meta-analysis

## Abstract

**Background:** It is widely accepted and acknowledged that data harmonization is crucial: in its absence, the co-analysis of major tranches of high quality extant data is liable to inefficiency or error. However, despite its widespread practice, no formalized/systematic guidelines exist to ensure high quality retrospective data harmonization.

**Methods:** To better understand real-world harmonization practices and facilitate development of formal guidelines, three interrelated initiatives were undertaken between 2006 and 2015. They included a phone survey with 34 major international research initiatives, a series of workshops with experts, and case studies applying the proposed guidelines.

**Results:** A wide range of projects use retrospective harmonization to support their research activities but even when appropriate approaches are used, the terminologies, procedures, technologies and methods adopted vary markedly. The generic guidelines outlined in this article delineate the essentials required and describe an interdependent step-by-step approach to harmonization: 0) define the research question, objectives and protocol; 1) assemble pre-existing knowledge and select studies; 2) define targeted variables and evaluate harmonization potential; 3) process data; 4) estimate quality of the harmonized dataset(s) generated; and 5) disseminate and preserve final harmonization products.

**Conclusions:** This manuscript provides guidelines aiming to encourage rigorous and effective approaches to harmonization which are comprehensively and transparently documented and straightforward to interpret and implement. This can be seen as a key step towards implementing guiding principles analogous to those that are well recognised as being essential in securing the foundational underpinning of systematic reviews and the meta-analysis of clinical trials.

## Introduction

Collaborative research programmes co-analysing individual participant data across studies are central to contemporary health science. The rationales underpinning such an approach include ensuring: sufficient statistical power; more refined subgroup analysis; increased exposure heterogeneity; enhanced generalizability and a capacity to undertake comparison, cross validation or replication across datasets.^1–3^ Integrative agendas also help maximizing the use of available data resources and increase cost-efficiency of research programmes.^1^^,^^4^

Co-analysis of data across multiple studies can be achieved in several ways, including: study-specific data analysis (independent analysis-by-study followed by meta-analysis of study-level estimates); pooled data analysis (data transferred to a central server and analysed as a collective whole); and federated data analysis (centralized analysis, but the individual-level participant data remain on local servers).^5^^,^^6^ However, to ensure content equivalence across studies and minimize measurement/assessment error that can cause bias or impair statistical power,^7^ all such approaches require use of harmonized data. Essentially, data harmonization achieves or improves comparability (inferential equivalence) of similar measures collected by separate studies.^8^

The use of compatible protocols to prospectively collect common measures undoubtedly facilitates harmonization.^9^ However, implementation of a prospective approach is not always possible or suitable. Repeating identical protocols is not necessarily viewed as providing evidence as strong as that obtained by exploring the same topic but using different designs and measures. In addition, investigators often need, for technical or scientific reasons, to use study-specific data collection devices. Finally, it is almost impossible to foresee all future harmonization requirements when implementing a new study. Retrospective harmonization (i.e. harmonization after data collection) is thus often the only option to permit data integration.^10^ Retrospective approaches have supported numerous, relatively small^11–15^ as well as very large research programmes.^16–21^ For instance, international human immunodeficiency virus (HIV) research networks^22–24^ that integrate existing HIV-related data are crucial to support current and upcoming research needs and develop appropriate health policies in the field. However, the increasing number of such programmes stresses an imperative to ensure quality, reproducibility and transparency of the results produced.

In systematic reviews and meta-analyses, the validity of a review depends on the use of a rigorous and transparent methodology.^25^ Whereas traditional or narrative reviews are useful when conducted properly, it is recognized that they can sometimes be of poor quality, biased or lead to inappropriate recommendations.^25^ In the past decades, guidelines for the conduct and reporting of systematic reviews and meta-analyses have therefore been articulated and consistently updated by consensus of experts.^26–28^ Such guidelines identify and provide a rationale for the steps required to conduct a rigorous review and are considered compulsory in preparing formal review articles. Ensuring the reproducibility and validity of harmonized data also demands rigorous procedures, which must be transparent if they are to be accepted as valid. However, because no systematic guidelines are currently available, most investigators harmonizing data ‘learn the hard way’: repeatedly encountering significant pitfalls. Reports on retrospective harmonization procedures applied by research networks have been published,^13^^,^^20^^,^^29^ and recently Rolland *et al.* described the process used at the Fred Hutchinson Cancer Research Center.^30^ Although the paper provides a useful high -level overview of an approach comparable to the one we foster, it does not address the details of the component elements we developed in the past decade to formally underpin a generic harmonization guidelines applicable across disciplines. Nevertheless, Rolland’s paper concurs with us that many researchers fail to reliably record basic information about the procedures used, decisions made and challenges encountered during the harmonization process and stresses the need to promote the creation of common and rigorous approaches to harmonization. Such guidance is essential for investigators new to the field to get to know issues to be addressed, and for groups reporting on their experience to identify the critical information to be made available if others are to properly estimate the quality of their work and learn from the successes and pitfalls they encountered.

The present paper provides an overview of the profile of key international initiatives and the approaches they use to harmonize data. It also details the guidelines developed in the past decade by Maelstrom Research and its partners, through a series of iterative reviews, consensus meetings and piloting within different harmonization programmes. The underlying goals of the guidelines are to foster a generic, but systematic, approach to retrospective data harmonization, and provide methodological guidance for investigators achieving harmonization and integration of pre-existing data. Detailed information and procedures are provided in supplementary materials, available as Supplementary data at *IJE* online.

## Methods

The guidelines proposed are the results of three integrated activities carried out from 2006 to 2015. These comprise: a phone survey with major international initiatives to gather a clear overview of the current retrospective harmonization practices; formal workshops with experts to build the guidelines and overview its iterations; and a series of case studies to evaluate and pilot different iterations of the guidelines.

### Exploring current practices

**Figure 1:**
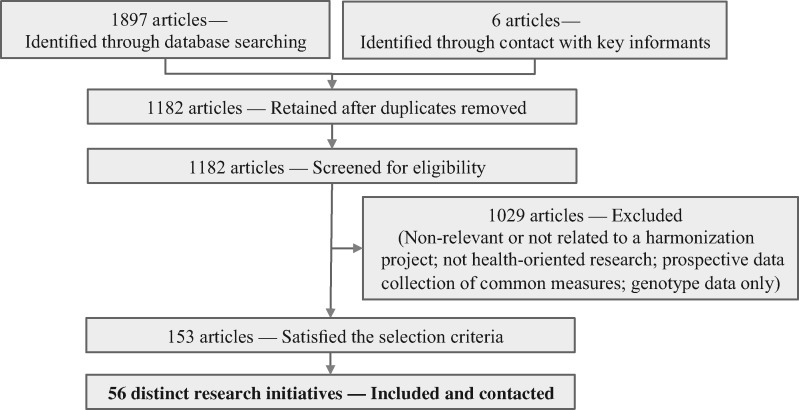
Flow chart describing selection of harmonization initiatives from literature search and references from key informants.

A literature search supplemented by references from key informants helped to identify initiatives having retrospectively harmonized individual participant’s data across epidemiological studies (Figure 1). Research initiatives were selected instead of specific papers because most challenges faced and methods used ought logically to remain comparable across a given project, even if several publications are generated.

A literature search was undertaken in Medline^®^, EMBASE^®^, PsycINFO^®^ databases and Google search engine using a range of keywords including ‘harmonization, pooled analysis, multiple studies consortium and meta-analysis’. The search was supplemented by a review of the articles cited in the selected papers and references from key informants. Articles identified were defined as eligible if they were published from January 2000 to March 2014 and reported results from initiatives having: achieved retrospective harmonization and integration of individual participant data; integrated data from at least two epidemiological studies; and analysed data on risk factors and health outcomes.

A total of 1182 articles were retrieved after removal of duplicates. Screening of titles and abstracts led to the identification of 153 articles satisfying all inclusion criteria. From those articles, 56 distinct initiatives conducting retrospective harmonization were identified and included in the survey. For each initiative, a key respondent was contacted by e-mail and, if not answering, re-contacted at least once by e-mail and once by phone to ask for participation. A semi-structured questionnaire was addressed to respondents agreeing to participate (lead investigators or a member of the research team responsible for data harmonization). The questionnaire addressed the aims, characteristics and infrastructure of the project, steps and methods applied to conduct the harmonization process, tools used and challenges faced. Descriptive analyses were conducted to explore the responses and compare characteristics of the participating and non-participating initiatives.

### Developing and piloting of the guidelines

A series of international workshops were organized to gather input from experts and examine different iterations of the guidelines. More than 100 investigators from a variety of backgrounds (epidemiologists, computer scientists, statisticians, ethicists, data librarians, etc.), research interests (research on ageing, twins, cancer, diabetes, etc.) and over 15 countries provided input. Using an iterative review and consensus approach, a subgroup of core investigators brought together the results gathered through these meetings, established guiding principles and developed the Maelstrom Research guidelines. Iterative versions of the guidelines were produced and tested within a series of harmonization projects: Promoting Harmonisation of Epidemiological Biobanks in Europe;^31^ Public Population Project in Genomics and Society;^32^^,^^33^ Canadian Partnership for Tomorrow Project;^34^ and Biobank Standardisation and Harmonisation for Research Excellence in the European Union.^35^ More recently, the Biobanking and Biomolecular Resources Research Infrastructure–Large Prospective Cohorts^36^ and the InterConnect project^37^ also applied the guidelines proposed.

## Results

Among the 56 study representatives contacted, 34 (60.7%) responded to the survey, two (3.6%) declined participation and 20 (35.7%) did not reply after three contacts. General characteristics of the 34 participating initiatives are presented in Table 1. A majority of the initiatives (*N* = 25; 73.5%) consisted of large consortia or collaborative networks addressing various research questions or generating harmonized datasets to serve longer-term goals; and 19 (55.9%) harmonized data only from studies of similar designs (e.g. all cohorts). Projects integrating data from multiple countries represented 76.5% (*N* = 26) of the initiatives. The number of individual studies within each initiative varied from 2 to 121, half of the initiatives (*N* = 18; 52.9%) harmonizing data from more than 10 studies. As for the total number of participants, 13 initiatives (38.2%) integrated data from more than 100 000 individuals, 15 (44.1%) from 10 000 to 100 000 individuals and six (17.6%) from less than 10 000 individuals. No differences were observed when the research areas, harmonization approaches or specific characteristics of the participating initiatives were compared with the non-participating initiatives (results not shown).

**Table 1.** General characteristics of the harmonization initiatives surveyed

Initiative (ref)	Countries	Number of studies	Study designs	Main topics
AirPROM^39^^a^	International	4	Cohort;	Asthma and chronic pulmonary obstructive diseases
			Registry	
APCSC^40^	International	44	Cohort	Cardiovascular risk factors and stroke, coronary heart disease and total cardiovascular diseases
BioSHaRE^41^	International	8	Cohort;	Metabolic risk factors and obesity
			Cross-sectional	
CHANCES^42^^a^	International	15	Cohort; Repeated cross-sectional	Cardiovascular diseases, diabetes mellitus, cancer, fractures and cognitive impairment
CLESA^11^	International	6	Cohort	Predictors of institutionalization, hospitalization and mortality
CLOSER^43^^a^	UK	9	Cohort; Panel	Broad topics (interdisciplinary research across longitudinal studies)
COSMIC^44^	International	19	Cohort	Cognitive measures and dementia
DYNOPTA^45^	Australia	9	Cohort	Cognitive measures, dementia and functional disabilities
ENGAGE^46^	International	36	Cohort;	Cardiometabolic traits
			Cross-sectional	
ENRIECO^20^	International	19	Cohort	Environmental risk factors in pregnancy and early childhood
EPIC^47^	International	23	Cohort	Cancer and chronic diseases
EPOSA^13^	International	5	Cohort	Osteoarthritis
ERFC^17^	International	121	Cohort	Cardiovascular risk factors
EURALIM^21^	International	7	Cross-sectional	Diet and cardiovascular risk factors
GENEVA^29^	International	16	Observational study not specified;	Genetic and environmental risk factors for health and disease
			Clinical trial/intervention trial	
GenomEUtwin^48^	International	8	Registry	Genetic and environmental risk factors for health and disease
HALCyon^49^	UK	9	Cohort	Physical capabilities
IALSA^50^	International	60	Cohort	Cognitive and physical capabilities, health, personality and well-being
INHANCE^51^	International	35	Case-control	Head and neck cancer
IDEFICS^12^	International	7	Cohort;	Childhood obesity
			Cross-sectional	
IPD Meta-Analysis^52^	Canada	3	Cohort;	Cognitive measures
			Cross-sectional	
LASA and NLSAA^53^	International	2	Cohort	Methodological differences in the harmonization of two longitudinal studies
MAGGIC^54^	International	31	Observational study not specified;	Survival of patients with heart failure with preserved or reduced left ventricular ejection fraction
			Clinical trial/intervention trial	
MeRGE^55^	International	30	Case-control; Nested case-control	Restrictive diastolic filling pattern and mortality in patients post-acute myocardial infarction and patient with chronic heart failure
MORGAM^56^	International	28	Cohort;	Cardiovascular risk factors and outcomes
			Repeated cross-sectional	
PAGE^57^	USA	8	Cohort;	Genetic and environmental risk factors for health and disease
			Cross-sectional;	
			Nested case-control;	
			Clinical trial/intervention trial	
PROG-IMT^58^	International	50	Cohort; clinical trial/intervention trial	Cardiovascular events and carotid intima-media thickness
PPPSDC^16^	International	28	Case-control	Diet and cancer
PPSRH^59^	International	12	Cross-sectional	Self-rated health
RELATE^60^^a^	International	14	Cross-sectional; Panel	Early life conditions and older adult health
THLS^61^^a^	Finland	3	Cohort;	Harmonization of clinical data between three studies
			Cross-sectional	
TLCS and HPHS^62^	USA	2	Cohort	Personality and health
TSC^63^	International	11	Cohort	Hypothyroidism, coronary heart disease and mortality risk
xTEND^64^	Australia	2	Cohort	Health and well-being

^a^ This information was gleaned from the initiative’s website or sources other than published articles.

AirPROM, Airway Disease Predicting Outcomes through Patient Specific Computational Modeling; APCSC, Asia Pacific Cohort Studies Collaboration; BioSHaRE, Biobank Standardisation and Harmonisation for Research Excellence in the European Union; CHANCES, Consortium on Health and Ageing: Network of Cohorts in Europe and in the USA; CLESA, Comparison of Longitudinal European Studies on Aging; CLOSER, Cohort & Longitudinal Studies Enhancement Resources; COSMIC, Cohort Studies of Memory in an International Consortium; DYNOPTA, Dynamic Analyses to Optimise Ageing; ENGAGE, European Network for Genetic and Genomic Epidemiology; ENRIECO, European initiative Environmental Health Risks in European Birth Cohorts; EPIC, European Prospective Investigation into Cancer and Nutrition; EPOSA, European Project on Osteoarthritis; ERFC, Emerging Risk Factor Collaboration; EURALIM, EURope ALIMentation; GENEVA, Gene Environment Association Studies; GenomEUtwin, GenomEUtwin; HALCYon, Health Ageing across the Life Course; IALSA, Integrative Analysis of Longitudinal Studies on Aging; IDEFICS, Identification and prevention of Dietary and lifestyle-induced health Effects In Children and infants; INHANCE, International Head and Neck Cancer Epidemiology; IPD Meta-Analysis, Harmonization of Cognitive Measures In IPD meta-analysis; LASA and NLSAA, Longitudinal Aging Study Amsterdam and Nottingham Longitudinal Study of Activity and Ageing; MAGGIC, Meta-analysis Global Group in Chronic Heart Failure; MeRGE, Meta-analysis Research Group in Echocardiography; MORGAM, MOnica Risk, Genetics, Archiving and Monograph; PAGE, Population Architecture using Genetics and Epidemiology; PROG-IMT, PROGression of Carotid Intima Media Thickness study; PPPSDC, Pooling Project of Prospective Studies of Diet and Cancer; PPSRH, Pooling Project on Self-Rated Health; RELATE, Research on Early Life and Aging Trends and Effects; THLS, National Institute for Health and Welfare (THL) studies (FINRISK cohorts, Health 2000 cohort and Helsinki Birth Cohort Study); TLCS and HPHLS, Terman Life Cycle Study and Hawaii Personality and Health Longitudinal Study; TSC, Thyroid Studies Collaboration; xTEND, eXtending Treatments, Education, and Networks in Depression study.

Infrastructures used to host and integrate data varied across initiatives. For the majority (*N* = 26; 76.5%), study-specific data were sent to a central location to permit integration and analysis. However, five (14.7%) initiatives included studies restricting data transfer, so data remained on study-specific servers. Three (8.8%) projects included some studies for which data were sent centrally and others in which they were hosted locally. When data were hosted locally, the harmonization process was generally rendered possible by sending the studies ready-to-use scripts to generate the harmonized variables and undertake a statistical analysis. Results generated were then combined using meta-analysis. However, two projects used a federated approach to remotely harmonize and analyse data hosted locally. Harmonization and processing were mainly achieved with regular statistical software (*N* = 31; 91.2%), except for three initiatives that used specialized software developed to support harmonization. As for data processing, algorithmic transformations (e.g. recoding of categories) was applied by all initiatives, and statistical models (e.g. regression analysis with standardized methods) were used by more than half (*N* = 23; 67.6%).

Respondents were asked to delineate the specific procedures or steps undertaken to generate the harmonized data requested. Sound procedures were generally described; however, the terminologies, sequence and technical and methodological approaches to these procedures varied considerably. Most of the procedures mentioned were related to defining the research questions, identifying and selecting the participating studies (generally not through a systematic approach), identifying the targeted variables to be generated and processing data into the harmonized variables. These procedures were reported by at least 75% of the respondents. On the other hand, few reported steps related to validation of the harmonized data (*N* = 4; 11.8%), documentation of the harmonization process (*N* = 5; 14.7%) and dissemination of the harmonized data outputs (*N* = 2; 5.9%).

A consensus approach was used to assemble information about pitfalls faced during the harmonization process (Box 1), establish guiding principles and develop the guidelines. The iterative process (informed by workshops and case studies) permitted to refine and formalize the guidelines. The only substantive structural change to the initial version proposed was the addition of specific steps relating to the validation, and dissemination and archiving of harmonized outputs. These steps were felt essential to emphasize the critical nature of these particular issues.

The guidelines proposed include a series of essentials compulsory to the success of data harmonization (

Box 1. Overview of the potential pitfalls in data harmonization identified by the respondents and experts

- ensuring timely access to data;
- handling dissimilar restrictions and procedures related to individual participant data access;
- managing diversity across the rules for authorship and recognition of input from study-specific investigators;
- mobilizing sufficient time and resources to conduct the harmonization project;
- gathering information and guidance on harmonization approaches, resources and techniques;
- obtaining comprehensive and coherent information on study-specific designs, standard operating procedures, data collection devices, data format and data content;
- understanding content and quality of study-specific data;
- defining the realistic, but scientifically acceptable, level of heterogeneity (or content equivalence) to be obtained;
- generating effective study-specific and harmonized datasets, infrastructures and computing capacities;
- processing data under a harmonized format taking into account diversity of: study designs and content, study population, synchronicity of measures (events measured at different point in time or at different intervals when repeated) etc;
- ensuring proper documentation of the process and decisions undertaken throughout harmonization to ensure transparency and reproducibility of the harmonized datasets;
- maintaining long-term capacities supporting dissemination of the harmonized datasets to users.

Box 2) and espouse an iterative process composed of a series of closely related and interdependent steps. An overview of the steps is provided below, but a comprehensive and structured description is presented as supplementary material. The Supplementary Material (available as Supplementary data at *IJE* online) lists, for each step and sub-step, The specific: aim; rational; procedures to be applied to ensure systematic process; issues to consider; resources that can be useful to facilitate the process; outputs generated; and an illustrative example. A checklist helping investigators to oversee the harmonization process is provided in Table 2.

**Table 2.** Checklist helping to review the harmonization process

Step	Item	Description
Step 0: define the questions and objectives	1	The research question is well defined in term of population, exposure, comparator, outcome and timing
	2	The protocol takes into account questions related to feasibility (e.g. data access, realistic time-lines) and provides information required to guide the harmonization process
Step 1: assemble information and select studies		
Step 1a: document individual study designs, methods and content	3	Study-specific information gathered allows understanding study designs, time-line, population characteristics, data contents, standard operating procedures and ethico-legal requirements to access data
Step 1b: select participant studies	4	Studies are selected based on explicit selection criteria
Step 2: define variables and evaluate harmonization potential		
Step 2a: select and define the core variables to be harmonized (DataSchema)	5	The DataSchema variables are selected based on their relevance in answering the research question addressed, likelihood to be generated across a number of studies and, where relevant, input from experts
	6	The DataSchema variables are clearly defined, including their specific nature, format and acceptable level of heterogeneity
Step 2b: determine the potential to generate the DataSchema variables making use of study-specific data items	7	The potential (or not) for each study to create the DataSchema variables is assessed and documented
Step 3: process data		
Step 3a: ensure access to adequate study-specific data items and establish the overall data processing infrastructure	8	If harmonization is possible, the study-specific data items required to generate the DataSchema variables are made available in a computing infrastructure allowing data processing
	9	Quality of study-specific data items is assessed and considered adequate
Step 3b: process study-specific data items under a common format to generate the harmonized dataset(s)	10	Data processing is achieved using appropriate statistical models or processing algorithms
	11	Harmonized data are generated and algorithms or models used to process data are documented
Step 4: estimate quality of the harmonized dataset(s) generated	12	Quality and consistency of the harmonized data are assessed. Where appropriate, statistical models are applied to evaluate heterogeneity and potential bias
Step 5: disseminate and preserve final harmonization products	13	Harmonized data are available to approved users
	14	All information required to understand harmonization procedures and to analyse the harmonized data are accessible

### Iterative steps toward data harmonization (see also Supplementary Material

Step 0: *Define the questions, objectives and protocol: develop a protocol reflecting the potential and limitations of the project. To ensure feasibility and reproducibility and to guide rational decision making, the objectives and research protocol must be clearly defined.*

Step 1: *Assemble information and select studies.*

Step 1a: *Document individual study designs, methods and content: ensure appropriate knowledge and understanding of each study. Data comparability can be affected by heterogeneity of study-, population-, procedural- and data-related characteristics. Information related to design, time frame and population background will, for example, be required to evaluate study eligibility. In addition, information related to the specific data collected and, where relevant, standard operating procedures used will be essential to evaluate harmonization potential and guide data processing.*

Step 1b: *Select participant studies: select studies based on explicit criteria. To ensure consistency, designs of the studies included in a harmonization project must be similar enough to be considered compatible.*

Step 2: *Define variables and evaluate harmonization potential.*

Step 2a: *Select and define the core variables to be harmonized: outline the set of outcome, exposure and confounding variables that will serve as reference- or target- for the harmonization of study-specific data items and will serve to answer the research questions addressed (i.e. the DataSchema).^38^ The nature of the DataSchema variables should reflect a satisfactory balance between targeting very precise concepts (e.g. identical questions) that optimize homogeneity, and acceptance of a greater degree of heterogeneity permitting inclusion of a larger number of studies. Explicit delineation and documentation are essential to inform the scientific meaning of the DataSchema variables and facilitate proper decision making throughout the harmonization process. For example, the definition of the variable ‘participant weight’ should include its units (kg) and a record of the decision to accept (or not) both measured and self-reported weights. In many settings it is also crucial to define temporal proximity with other information of interest (e.g. collections of weight and physical activity).*

Step 2b: *Determine the potential to generate the core (DataSchema) variables making use of study-specific data items: determine whether each study can construct-or not-each of the DataSchema variables as defined. It is necessary to evaluate which studies can provide data that enable generation of each of the DataSchema variables and to qualitatively assess the level of similarity between the study-specific and DataSchema variables. For example, only studies that measure participant weights could be viewed as being able to create the DataSchema variable ‘Measured participant weight’.*

Step 3: *Process data.*

Step 3a: *Ensure access to adequate study-specific data items and establish the overall data processing infrastructure: ensure accessibility to, and quality of, the study-specific data items required to create the harmonized dataset. To allow data processing, it is essential to ensure availability and quality of all relevant study-specific data items. It is also a prerequisite to implement a data-processing infrastructure adapted to the context of the project and level of access to information allowed (access to individual participants’ data, or access restricted to aggregated data or study-level results of statistical analysis) (Table 3). The data processing infrastructure will comprise both the study-specific (input data) and harmonized data generated (output data).*

Box 2. Absolute essentials required to achieve any successful harmonization project

*Collaborative framework:* a collaborative environment needs to be implemented to ensure the success of any harmonization project. Investigators involved should be open to sharing information and knowledge, and investing time and resources to ensure the successful implementation of a data-sharing infrastructure and achievement of the harmonization process.

*Expert input:* adequate input and oversight by experts should be ensured. Expertise is often necessary in: the scientific domain of interest (to ensure harmonized variables permit addressing the scientific question with minimal bias); data harmonization methods (to support achievement of the harmonization procedures); and ethics and law (to address data access and integration issues).

*Valid data input*: study-specific data should only be harmonized and integrated if the original data items collected by each study are of acceptable quality.

*Valid data output*: transparency and rigour should be maintained throughout the harmonization process to ensure validity and reproducibility of the harmonization results and to guarantee quality of data output. The common variables generated necessarily need to be of acceptable quality.

*Rigorous documentation:* publication of results generated making use of harmonized data must provide the information required to estimate the quality of the process and presence of potential bias. This includes a description of the: criteria used to select studies; process achieved to select and define variables to be harmonized; procedures used to process data; and characteristics of the study-specific and harmonized dataset(s) (e.g. attribute of the populations).

*Respect for stakeholders:* all study-specific as well as network-specific ethical and legal components need to be respected. This includes respect of the rights, intellectual property interests and integrity of study participants, investigators and stakeholders.

Box 3. Examples of data processing models

*Algorithmic transformation*: Continuous and categorical variables, or both, with different but combinable ranges or categories (e.g. education level, household income)

*Simple calibration model*: Continuous metrics with calibration model (e.g. weight in kilograms or pounds)

*Standardization model*: Continuous constructs measured using different scales, with no known calibration method or bridging items (e.g. two independent memory scales)

*Latent variable model*: Continuous constructs measured using different scales, with no known calibration method but with bridging items (e.g. two memory scales, with some common items)

*Multiple imputation models*: Continuous or categorical constructs measured using overlapping scales permitting imputation of missing values (e.g. two overlapping scales measuring activities of daily living)

**Table 3.** Impact of the level of information that is available from each study on the harmonization process

Level of information available	Location of study-specific individual participant data	Achievement of data processing	Application of the processing models (see Box 3)
Individual participant data	Transferred on a central server or remain on individual study’s servers	Generally achieved centrally	All models
Aggregated data (e.g. means and frequencies)	Remain on individual study’s servers	Achieved by study-specific teams. Can be centralized if a federated infrastructure is implemented	Limited to some models
Final results of statistical analysis	Remain on individual study’s servers	Achieved by study-specific teams	Limited to some models

Step 3b: *Process study-specific data under a common format to generate the harmonized dataset(s): convert the heterogeneous study-specific data items to DataSchema variables. Data processing is achieved using algorithms recoding study-specific data or statistical models based on contemporaneous analysis (Box 3). The procedures adopted will depend on the nature and format of the variables and on the data-processing infrastructure implemented.*

Step 4: *Estimate quality of the harmonized dataset(s) generated: understand the characteristics and utility of the harmonized dataset(s) generated. In order to ensure statistical analyses are run on data of acceptable quality, quality control procedures must be undertaken. The procedures should include verification of the algorithms or statistical models applied, and generation of basic quality checks and descriptive statistics (to evaluate consistency of the harmonized data across studies and explore potential influence of bias). When possible, procedures should be applied to test harmonization assumptions and assess heterogeneity.*

Step 5: *Disseminate and preserve final harmonization products: implement a sustainable infrastructure to preserve and disseminate harmonized data. In order for investigators not directly involved in the harmonization process to understand the steps and decisions taken, access to appropriate documentation must also be provided. This should include variable-specific metadata (e.g. harmonization potential, algorithms or statistical model used to process data) and description of the harmonization procedures applied. Ideally, all data and metadata should be made available in standard formats.*

## Discussion

Achieving retrospective harmonization is necessarily challenging. This is particularly true for multidisciplinary initiatives like the ALPHA network (Analysing the Longitudinal Population-based HIV/AIDS data in Africa), including researchers from a variety of disciplines aiming to answer a broad range of research questions. Data harmonization is time consuming, and demands significant technical and scientific investments. Adding to the hurdle, harmonization is context-specific and the process generally needs to be repeated if new scientific questions arise. Furthermore, investigators need to ensure that data is only claimed to be harmonized if the process generated common variables of acceptable quality. Fortunately, a number of factors can facilitate the process and increase cost-effectiveness. For example, working within networks open to collaboration will facilitate sharing of data, resources and knowledge (Step 0). The identification of studies of interest (Step 1) and evaluation of the harmonization potential (Step 2) are facilitated by the existence of central metadata catalogues providing comprehensive information on existing study designs and content. Catalogues can also provide information useful to guide the development of prospective data collections. Data processing (Step 3) and the dissemination and preservation of the harmonized datasets (Step 5) are facilitated by usage of specialized software offering a secure, scalable and cost-effective computing environment. Access to comprehensive documentation about past harmonization initiatives can inform investigators about suitable processing models (Step 3) and quality control procedures (Step 4) and simplify achievement of harmonization in new, but similar contexts. Finally (Step 5), providing timely, appropriately governed access to harmonized datasets^38^ helps to ensure effective return on the investments made and can act as a springboard to a wide range of additional research activities.

It is acknowledged that harmonization is important, requires thorough preparatory work, and has many elements that must be worked through carefully and systematically. However, many of the key steps to harmonization appear self-evident and straightforward even if time consuming to carry out. As a result, harmonization is often seen as a task that can easily be undertaken even by an ‘enthusiastic amateur’. This precisely reflects early perspectives on systematic reviews, meta-analyses and clinical trials before formal guidelines and protocols were accepted as the norm. Unfortunately, no matter that many harmonization efforts are of high quality, the lack of collectively agreed terminologies and guidelines or protocols - emphasizing both documentation and quality control - makes it almost impossible for others to learn from those with practical experience, or even to objectively decide whether a particular harmonization project has been done well. To descend into cliché: reinvention of the wheel is all too common and, more seriously, the invention of non-functional wheels (e.g. with a missing axle) is far from rare. Virtually nobody with knowledge and experience in contemporary health science would argue that it would be preferable to undertake a clinical trial, a systematic review or a meta-analysis-particularly a first foray into any of these activities without following accepted guidelines. This ensures that no critical steps are missed, everything that others might later view as crucial information is properly documented and appropriate quality assurance criteria are evaluated. It is a central message of the current paper that harmonization should be viewed in precisely the same way and is the reason why we outlined these guidelines. Building robustly on the more detailed thinking laid out in supplementary materials (available as Supplementary data at *IJE* online), these guidelines have been applied to, and developed across, a number of harmonization initiatives that we believe have been successful. With this as a starting point, we encourage the scientific community, journal editors and funding agencies to debate and refine these guidelines with the aim of collectively agreeing on a generic protocol for data harmonization. Once this has been agreed, the harmonization procedures adopted in preparing a set of observational epidemiological studies for joint analysis can be held up to scrutiny against agreed best practice. Only then will harmonization initiatives - like systematic reviews, meta-analyses and clinical trials - be reliably undertaken in an effective manner, and will such initiatives be properly evaluated in judging grant applications, reviewing papers or interpreting the published literature.

## Supplementary data

Supplementary data are available at *IJE* online.

## Funding

This work was supported by funds from the Quebec ‘Ministère de l’Enseignement supérieur, de la Recherche, de la Science et de la Technologie’; the Canadian Partnership against Cancer, the European Union’s Seventh Framework HEALTH-F4-2010 grant 261433 (BioSHaRE.eu); the Canadian Longitudinal Study on Aging, funded by the Canadian Institutes of Health Research and the Canadian Foundation for Innovation; the Ontario Institute for Cancer Research through funding provided by the Government of Ontario, Canada; and the Genome Canada and Genome Quebec funding agencies. The D2K (Data to Knowledge) programme of methods research in infrastructural epidemiology at the University of Bristol is supported by joint awards from the MRC and Wellcome Trust underpinning the ALSPAC project and the Biomedical Resource of the 1958 Birth cohort; MRC funding for the Welsh and Scottish Farr Institutes; ESRC funding of the CLOSER initiative and the BBMRI-LPC (Biobanking and Biomolecular Resources Research Infrastructure-Large Prospective Cohorts EU FP7, I3 grant). This work is also supported by a Tier 1 Canada Research Chair in GeroScience; and the Raymond and Margaret Labarge Chair in Research and Knowledge Application for Optimal Aging.

Key Messages

- A wide variety of initiatives use retrospective data harmonization as a keystone of their research work.
- Even when appropriate approaches are used, the terminologies, procedures, technologies and methods used vary markedly across initiatives.
- Building on the combined findings of a phone survey, expert workshops and case studies, we have developed, and here report, formal guidelines for retrospective harmonization comprising a series of essentials and interactive steps.
- The guidelines aim to encourage rigorous and effective approaches to harmonization, which are comprehensively and transparently documented and straightforward to interpret and implement.

## Supplementary Material

Supplementary Data
