# Supplementary material for: Maelstrom Research guidelines for rigorous retrospective data harmonization
Source: Int J Epidemiol. 2016 Jun 5;46(1):103–5. doi: 10.1093/ije/dyw075 (PMC5407152; doi:10.1093/ije/dyw075)
Supplement: Supplementary Data [file dyw075_supp.pdf]

SUPPLEMENTARY MATERIAL

MAELSTROM RESEARCH GUIDELINES FOR RIGOROUS RETROSPECTIVE DATA HARMONIZATION

Iterative steps toward data harmonization

Retrospective harmonization of individual participant data is an iterative process composed of a series of key closely related, inter-dependant and often integrated steps (Figure 1). Step 0 and 5 reflect the tasks to be completed pre- and post-harmonization, while Steps 1 to 4 represent the core process generating the harmonized data required for co-analysis. Achievement of these steps aims to: permit researchers to anticipate and cope with challenges to be faced and to structure decision making throughout the harmonization process. Please refer to the main paper for additional information.

Figure 1. Iterative steps toward data harmonization

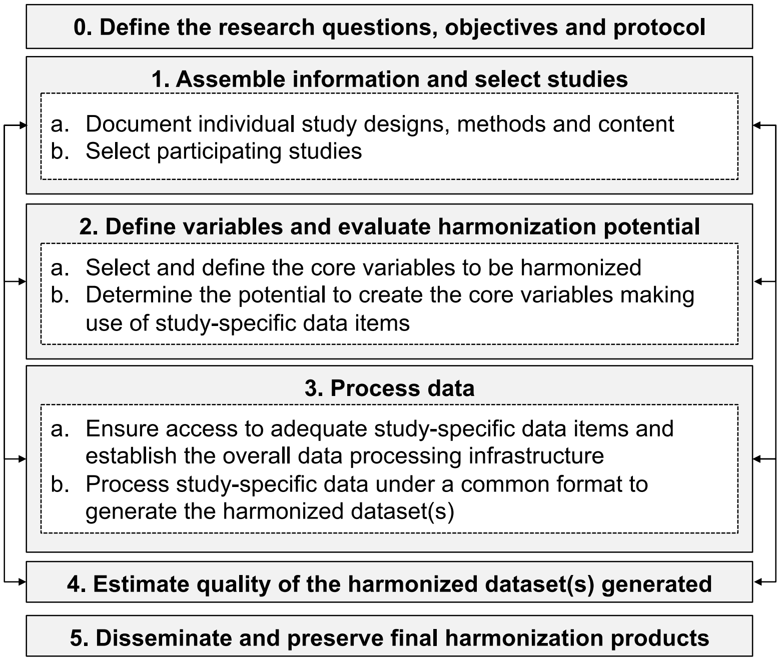

Step 0: Define the questions, objectives and protocol

Aim: Develop a protocol reflecting the potential and limitations of the research project.

Rationale: To ensure feasibility and reproducibility and to guide decision making, the objectives and scientific questions to be addressed must be clearly defined and a realistic protocol must be developed prior to beginning the harmonization process.

Procedures:

- Conduct a literature review on the research questions and gather, where required, scientific, methodological, ethico-legal and administrative input from experts.
- Define research questions and hypotheses in terms of Population, Exposures (or Intervention), Comparator, Outcome, and Timing (for longitudinal or intervention studies)[1]. This includes, but is not limited to, the definition of acceptable designs for the potential participating studies (study selection criteria) and the type of information required to answer the research question addressed (e.g. participant history of diabetes, socio-economic status).
- Define the data infrastructure to be implemented, including details related to the location of study-specific and harmonized data during the harmonization process.
- Develop the research protocol addressing ethical and legal matters and defining procedures, methods, budget, deliverables, timelines and infrastructure.

Issues to consider:

- As a number of individual studies will be involved, it is essential to consider study-specific as well as common issues in the development of the protocol and the collaboration agreements (e.g. operational or ethico-legal requirements foreseen to permit data integration across jurisdictions). Issues related to the "ownership" of the harmonized datasets generated should also be addressed.
- Questions related to study-specific data access and usage will influence the harmonization process and must be addressed. For example, will individual participant data be transferred to a central repository or will it remain on study-specific servers?

[1] Glasziou PP, Sanders SL. Investigating causes of heterogeneity in systematic reviews. Stat Med. 2002;21(11):1503-1511

- Responsibilities of study-specific investigators and core researchers implementing the harmonization process must be defined. For example, will the harmonization process be achieved by a central team or by the study-specific teams? A centralized process would certainly speed-up and increase the flexibility and standardization in decisions making, but will require close collaboration with studies to ensure proper understanding of data.

Helpful resources:

- Dynamic research network open to collaboration.
- Accessible knowledge on the technical, scientific and ethico-legal issues related to harmonization.
- Accessible repository permitting to evaluate feasibility of the research project (e.g. catalogue providing information about samples an data available as well as data sharing potential).

Output: Research protocol.

Example:

A series of specific research questions were defined and a protocol developed for the project: "Prevalence of obese individuals not showing increased metabolic or cardiovascular risk across biobanks participating to the BioSHaRE Healthy Obese project"[2].

[2] Doiron D, Burton P, Marcon Y, et al. Data harmonization and federated analysis of population-based studies: the BioSHaRE project. Emerg Themes Epidemiol. 2013;10(1):12

Step 1: Assemble information and select studies

Step 1a: Document individual study designs, methods and content

Aim: Ensure appropriate knowledge and understanding of each study.

Rationale: Data comparability can be affected by heterogeneity in study-, population- and data-related characteristics. Access to comprehensive information on study designs, population background, methods and data collected is thus essential to support the harmonization process and estimate potential sources of bias. Generic information related to design, time frame and population background will, for example, be required to evaluate study eligibility. In addition, comprehensive information related to the specific data collected and standard operating procedures used are essential to evaluate harmonization potential and support data processing.

Procedures:

- To guide study selection (step 1b), gather generic information on ethical and legal requirements, study designs, time frame, content, and population characteristics.
- To support evaluation of the harmonization potential and data processing (steps 2 and 3), gather comprehensive documentation on studies including, for example: research protocols, questionnaires, standard operating procedures, data dictionaries (codebooks), and procedures to access data.
- Assemble information within a functional repository.

Issues to consider:

- Collaborative projects can aim to harmonize a limited or a broad range of variables. Information to be collected can thus be restricted to specific domains (e.g. cancer history, depression) or cover a variety of information.

Helpful resources:

- Accessible repository providing comprehensive and standardized information on study designs and contents. If not available, financial and technical resources to gather and support studies providing the information required.

Outputs: Repository of information and documents required to support study selection and data harmonization process.

Example:

Study investigators and data custodians were contacted to gather comprehensive information on study designs (e.g. number of participants, selection criteria), questionnaires, standard operating procedures, data dictionaries, and procedures to access data.

Step 1b: Select participating studies

Aim: Select studies based on rigorous selection criteria.

Rationale: To ensure consistency, designs of the studies included in a harmonization project must be similar enough to be considered compatible. Conditions to be fulfilled by each study to be considered compatible must be clearly defined.

Procedures:

- Select participating studies using explicit eligibility criteria (e.g. twin studies including randomly selected twin pairs of at least 45 of age at recruitment and collecting at the same time point blood samples allowing extraction of cortisol level and information about anxiety levels).
- Create a list of participating studies.

Issues to consider:

- Definition of the eligibility criteria are specific to each harmonization project and will vary according to the questions and objectives defined in Step 0.
- Studies selected need to be sufficiently comparable to answer the research question addressed. For example, studies could need to use the same sampling frame (e.g. all population-based), recruit participants born at about the same historical time and use similar tools to collect data.
- To be eligible, a study does not necessarily need to permit the creation of *all* the harmonized variables targeted (Step 2).

Helpful resources:

- Accessible repository providing information about studies (e.g. basic study design, data collection timelines, number and characteristics of participants, original sampling frame, type of samples collected).

Output: List of participating studies responding to eligibility criteria.

Example:

Eight of the 14 population-based cohorts part of the BioSHaRE project were selected. To be eligible, studies needed to collect targeted health outcomes, socio-demographic, physical and biochemical measures on adult participants. Studies also needed to allow remote access to aggregate data for statistical analyses.

Step 2: Define variables and evaluate harmonization potential

Step 2a: Select and define the core variables to be harmonized

Aim: Outline the set of outcome, exposure and confounding variables which will serve as reference - or target - for the harmonization of study-specific data items (i.e. the DataSchema), and that will then be used to answer the research questions addressed.

Rationale: To ensure content equivalence - or adequate similarity - formal criteria permitting construction of each DataSchema variable need to be defined. Rigorous definitions are essential to outline the precise nature and scientific meaning of the harmonized variables to be created (common construct) and facilitate proper decision-making throughout the harmonization process.

Procedures:

- Based on scientific relevance, input from experts and exploration of data available across studies, identify the list of variables to be included in the DataSchema. For each variable, define the following attributes:

(1) provide a definition (e.g. participant weight) and specific characteristics such as format, categories, unit, etc. (e.g. unit=kg);
(2) identify conditions that could influence harmonization potential (e.g. self-reported weight versus measured weight);
(3) define the harmonization rules to be used to determine if a particular study can create the variable (e.g. it could be decided that self-reported measures of weight shouldn't be used to create the DataSchema variable); and
(4) define criteria to be used to assess precision of the DataSchema variable created or similarity between the study-specific and DataSchema variables (e.g. loss of precision if weight is collected as a categorical variable in some studies).

- Use an iterative process to ensure consensus on the variable definitions.

Issues to consider:

- Variable definitions should reflect a satisfactory balance between: (1) use of precisely uniform specifications (e.g. identical question or standard operating procedures) that render pooling straightforward but limit eligibility of studies; and (2) acceptance of some degree of heterogeneity enabling harmonization of similar but not necessarily identical data, and permitting inclusion of a larger number of studies.
- Variable definitions are context-specific and usually vary according to the research questions addressed. The acceptable balance of heterogeneity versus uniformity is influenced by factors such as the scientific objectives pursued, the availability of accepted scientific standards and the projected use of the harmonized variable. For example, investigators might consider using either reported or measured weights if weight is only

considered as a potential confounder, but might only include direct measurements if weight is a key outcome or a critical explanatory variable (e.g. determinants of type 2 diabetes).

- It will generally *not* be possible to create *all* DataSchema variables across all studies.

Helpful resources:

- Support from experts in specialized research areas (e.g. physical activity, depression, etc.) and data harmonization approaches.
- Access to information on comparable variables harmonized by other research groups, and on specific techniques used to harmonize the data.

Output: List of DataSchema variables and related attributes.

Example:

The DataSchema variables were selected by the project investigators at three consensus meetings. Each variable was defined as illustrated by the two examples below. Naturally, these definitions and requirements for homogeneity could have been very different in another scientific context.

Blood glucose level

Description: Laboratory measurement of participant fasting glucose level.

Format: Decimal. Unit: mmol/L.

Harmonization rules: (1) Participant must be fasting at least 8 hours before measurement, (2) all standard operating procedures used to generate measurements can be considered acceptable.

Occurrence of stroke

Description: Occurrence of stroke at any point during the life of the participant.

Format: No=Never had stroke; Yes=Has had stroke.

Harmonization rules: (1) Can be generated from a questionnaire to participants asking about the history of stroke (including or not cerebral haemorrhage or obstruction of a cerebral vessel), (2) can be generated from a questionnaire to participants asking for an open list of diseases, (3) question addressed must reflect all participant life and not be limited in time (e.g. in the last year), (4) questions allowing no distinctions between stroke and transitory ischemic attack should not be used to create the variable.

Step 2b: Determine the potential to generate the core variables (DataSchema) making use of study-specific data items

Aim: Determine the possibility for each study to construct each DataSchema variable.

Rationale: To ensure content equivalence it is necessary - before initiating data processing - to evaluate which studies can provide data allowing generation of the DataSchema variables and qualitatively assess the level of similarity between the study-specific and DataSchema variables.

Procedures:

- Evaluate and document the potential for each study to create each of the DataSchema variables by comparing: the list of study-specific data items collected, collection procedures and other relevant documentation obtained in Step 1, and the DataSchema variables definitions (Step 2a.).

- Estimate the level of similarity between the study-specific variables and DataSchema variables to be created.

Issues to consider:

- Generally, *all* studies will not enable the creation of *all* DataSchema variables identified.
- The evaluation of the potential to harmonize data often leads to revision and refinement of the DataSchema variables selection and definitions (Step 2a) and real potential to generate the DataSchema variable can only be confirmed following processing of study data (Step 3b).

Helpful resources:

- Accessible repository providing comprehensive and standardized information on study-specific content, including data dictionaries, questionnaires, and standard operating procedures.
- Accessible repository providing information on variables generated (e.g. definitions, statistical models or algorithms applied) and approaches used by past harmonization initiatives.

Output: Repository of information describing harmonization potential of each variable in each participating study.

Example:

A repository accessible to investigators was generated. Study specific data items and harmonization potential for the variables defined in the example of Step 2a are provided below.

Blood glucose level

Study A: Level of blood sugar (mmol/l) measured for non-fasting participants.

"Impossible Match"

Study B: Level of blood sugar (mmol/l) measured for fasting participants, but no specific question addressed to the participant to confirm time since the last meal.

"Possible Match (Partial)", possible to harmonize as the study protocol stated 8 hours fasting was required, but "partial as no confirmation of the non-fasting status for each participant.

Study C: Level of blood sugar (mmol/l) for fasting participants with specification on the time since the last meal.

"Possible Match"

Occurrence of stroke

Study A: Question: Have you ever been diagnosed with transitory ischemic attack or stroke? [Yes/No]

"Impossible Match"

Study B: Question: Has a doctor ever diagnosed you with stroke? [Yes/No].

"Possible Match"

Study C: Question: In the last 10 years, have you had a stroke? [Yes/No].

"Possible Match (Partial)", possible to harmonize, ten years considered as representative of "all life" but involves a loss of precision. Including *Study C* should lead to a modification of the DataSchema variable definition.

Step 3: Process data

Step 3a: Ensure access to adequate study-specific data items and establish the overall data processing infrastructure

Aim: Ensure accessibility and quality of the study-specific data items required to create the harmonized dataset.

Rationale: To permit data processing, it is essential to: ensure availability and quality of all the relevant study-specific data items required to generate the DataSchema variables and implement a data processing infrastructure adapted to the context of the harmonization project. The data processing infrastructure will comprise both the study-specific (input data) and harmonized data generated (output data).

Procedures:

- Identify the list of all study-specific data items required to construct the DataSchema variables (informed by Step 2b).
- Implement adequate data processing infrastructure.
- Ensure availability of the study-specific data under a manageable format.
- Assess quality of each study dataset and evaluate suitability of the study-specific data for processing (e.g. weight measure available for the majority of study participants and not including inconsistent values).

Issues to consider:

- The data processing infrastructure can be simple but can also be particularly complex and involve remote access to study-specific datasets.

- Obtaining all necessary approvals and abiding ethical and legal requirements of each study is a prerequisite to data access and implementation of the data infrastructure.
- As achieving procedures prerequisite to obtain data can take several months, these procedures must be achieved as early as possible in the harmonization process.

Helpful resources:

- Overarching or coordinated ethico-legal and governance policies across the targeted studies pertaining to data and samples usage and access.
- Proper computational capacities to set-up the required data processing infrastructures.

Outputs:

- Adequate study-specific datasets to be used to create the DataSchema variables.
- Descriptive statistics necessary to assess quality of study-specific data items.
- Data processing infrastructure adapted to the context of the harmonization project.

Example:

After obtaining approvals from all data access and ethical committees, secure servers were set-up in each study's host institution and the subsets of data required to generate target DataSchema variables were loaded onto each of these servers. For each study, data was checked for missing values and outliers. Data custodians were contacted when questions arose.

Step 3b: Process study-specific data under a common format to generate the harmonized dataset(s)

Aim: Create harmonized data (DataSchema) and generate a single pooled dataset or multiple independent study-specific dataset(s) to be used for data analysis.

Rationale: To permit valid co-analysis, it is necessary, where possible and scientifically appropriate (Step 2b), to convert the heterogeneous data items collected by individual studies to a set of harmonized (DataSchema) variables.

Procedures:

- When considered possible and scientifically relevant (Step 2b) process study-specific data under the DataSchema variables format. If required, achieve calibration analysis or develop complex statistical models.
- Ensure proper documentation of the process (e.g. data processing scripts, comments on specific decisions undertaken).
- Create one (or multiple study-specific) harmonized dataset(s) and associated comprehensive data dictionary.

Issues to consider:

- Data processing could be achieved using rule-based algorithms recoding study-specific data items; or statistical models based on contemporaneous analysis (e.g. to calibrate different study-specific anxiety scales).
- The procedures used to transform data will depend on the: research question addressed; nature and format of the DataSchema variables; nature and format of the study-specific data items; and data processing infrastructure implemented (Step 3a).

- Data processing can lead to revision and refinement of the DataSchema variables definitions.

Helpful resources:

- Specialized software and adequate data processing infrastructure allowing effective management of the data harmonization process.
- Knowledge on the technical, scientific issues related to data harmonization.
- Repository providing information on variables generated by past harmonization initiatives.
- Good support from study-specific data custodians or investigators with knowledge of the study.

Outputs: One (or several) harmonized dataset(s) and associated documentation.

Example:

Processing algorithms transforming study data into the DataSchema variable format were developed and implemented for each study whenever harmonization was deemed possible. Statistical models are also currently developed to harmonize some of the variables.

Step 4: Estimate quality of the harmonized dataset(s) generated

Aim: Understand characteristics and utility of the harmonized dataset(s) generated so as to ensure adequate usage.

Rationale: In order to ensure statistical analyses are run on data of acceptable quality, quality control procedures must be achieved and, where possible, heterogeneity of the harmonized datasets estimated.

Procedures:

- Ensure basic quality checks of the harmonized datasets. For example, formats (e.g. adequate categories created) and ranges of information (e.g. identification of outliers).
- Verify the algorithms or statistical model used match the study-specific to DataSchema variables.
- Run descriptive statistics to evaluate consistency of harmonized data across studies and explore the potential influence of bias.
- When possible, test harmonization assumptions and develop models to assess heterogeneity.

Issues to consider:

- Specific procedures used to test harmonization assumptions are context-dependent. For example, when using a statistical model to generate harmonized data, appropriateness of statistical processing could be assessed (e.g. interchangeability and invariance of the mathematical relation across subjects and studies). In addition, when using rule-based algorithms the impact of including/excluding particular studies presenting: (1) study-specific data items leading to loss of precision; or (2) specific characteristics that could be sources of bias (e.g. an unusual sampling frame) could be explored.
- Input from study data custodians is generally required to support the process and confirm the variables have been derived accurately.

Helpful resource: Accessible knowledge on quality control methods useful in the context of data harmonization.

Outputs: Final harmonized dataset(s) ready for statistical analysis.

Example:

Following validation of the algorithms applied, summary statistics (frequencies, median, mean and ranges) were compared across study-specific harmonized datasets and, where required, discussions with studies were engaged to understand potential causes of inconsistencies. Information about data quality was documented.

Step 5: Disseminate and preserve final harmonization products

Aim: Implement a sustainable infrastructure to preserve and disseminate harmonized data, variable-specific meta-data and documentation of the harmonization approach.

Rationale: In order for investigators not directly involved in the harmonization process to fully understand and/or evaluate the steps and decisions taken to produce the harmonized data, access must be provided to appropriate variable-specific meta-data and comprehensive documentation of the harmonization procedures applied.

Procedures:

- Implement structured archives permitting access to:
  a) DataSchema variables list and definitions (Step 2a)
  b) "Crosswalks" describing the specific relationships between the data items originally collected by studies and the DataSchema variables generated (Step 2b). This could include the algorithms or statistical model used to process data (Step 3b).
  c) Information about quality of the harmonized data (Step 4).
  d) Descriptive statistics extracted from the harmonized datasets (study-specific and pooled) (Step 4).

- Where required, implement a data infrastructure permitting long-term archiving, management and future access to the harmonized dataset(s) generated.

Issues to consider: Hosting centrally harmonized data generated from individual studies data items can raise specific ethical and legal issues. This includes, for example, the requirement for each study data access committee to grant - or not - access to the harmonized dataset(s) to external users.

Helpful resource: Access to recognized standards and tools to document and disseminate information about the harmonized dataset(s) generated and to manage harmonized data.

Outputs: Accessible repository of harmonized data, meta-data and related documentation.

Example:

A password protected web portal was implemented for the investigators to retrieve: (1) the list and definitions of the DataSchema variables generated, (2) the harmonization potential and processing rules applied to generate each DataSchema variable for each participating study, and (3) the study specific and harmonized data summaries (frequencies, min, max, mean, standard deviation).
